# A complex and punctate distribution of three eukaryotic genes derived by lateral gene transfer

**DOI:** 10.1186/1471-2148-7-89

**Published:** 2007-06-11

**Authors:** Matthew B Rogers, Russell F Watkins, James T Harper, Dion G Durnford, Michael W Gray, Patrick J Keeling

**Affiliations:** 1 Department of Botany, University of British Columbia, 3529-6270 University Boulevard, Vancouver, British Columbia V6T 1Z4, Canada; 2 Department of Biochemistry and Molecular Biology, Dalhousie University, Halifax, Nova Scotia, B3H 1X5, Canada; 3 Department of Biology, University of New Brunswick, Fredericton, NB, E3B 5A3, Canada; 4 Centre for Molecular Medicine and Therapeutics, 950 West 28th Avenue, Vancouver, B.C., V5Z 4H4, Canada

## Abstract

**Background:**

Lateral gene transfer is increasingly invoked to explain phylogenetic results that conflict with our understanding of organismal relationships. In eukaryotes, the most common observation interpreted in this way is the appearance of a bacterial gene (one that is not clearly derived from the mitochondrion or plastid) in a eukaryotic nuclear genome. Ideally such an observation would involve a single eukaryote or a small group of related eukaryotes encoding a gene from a specific bacterial lineage.

**Results:**

Here we show that several apparently simple cases of lateral transfer are actually more complex than they originally appeared: in these instances we find that two or more distantly related eukaryotic groups share the same bacterial gene, resulting in a punctate distribution. Specifically, we describe phylogenies of three core carbon metabolic enzymes: transketolase, glyceraldehyde-3-phosphate dehydrogenase and ribulose-5-phosphate-3-epimerase. Phylogenetic trees of each of these enzymes includes a strongly-supported clade consisting of several eukaryotes that are distantly related at the organismal level, but whose enzymes are apparently all derived from the same lateral transfer. With less sampling any one of these examples would appear to be a simple case of bacterium-to-eukaryote lateral transfer; taken together, their evolutionary histories cannot be so simple. The distributions of these genes may represent ancient paralogy events or genes that have been transferred from bacteria to an ancient ancestor of the eukaryotes that retain them. They may alternatively have been transferred laterally from a bacterium to a single eukaryotic lineage and subsequently transferred between distantly related eukaryotes.

**Conclusion:**

Determining how complex the distribution of a transferred gene is depends on the sampling available. These results show that seemingly simple cases may be revealed to be more complex with greater sampling, suggesting many bacterial genes found in eukaryotic genomes may have a punctate distribution.

## Background

Lateral gene transfer is the movement of genes between distantly related organisms, a phenomenon that has become a major focus in the study of genome evolution [1-7]. The importance of gene transfers between prokaryotic genomes is now generally recognized due to the many such genomes now available for comparison, although there is still controversy about how common prokaryote-to-prokaryote gene transfer is, and what long term effects it has [8-10]. For eukaryotes there are far fewer sequenced genomes to compare, and emerging evidence suggests that lateral gene transfer may not be prevalent in many of the lineages where the most data are available, such as vertebrates [11,12]. Nevertheless, convincing examples of prokaryote-to-eukaryote gene transfers have been described [13-17], and transfers between eukaryotes are also known [15,18-21]. While these studies make it clear that lateral transfer has affected eukaryotic nuclear genomes, the frequency of such events and the extent of their evolutionary impact, particularly for eukaryote-to-eukaryote lateral transfers remains unknown. In particular, most known cases involve genes moving from a prokaryote to eukaryotes, whereas comparatively little is known about transfers between eukaryotes.

Various approaches have been used to infer gene transfers between prokaryotic genomes [22]. By far the most common method of detecting events involving eukaryotes is to observe incongruence between phylogenetic trees based on a gene and the tree that is considered (on the basis of other evidence) to reflect the evolution of the organism in which the gene is encoded. Lateral gene transfer events are thus commonly invoked to explain trees that depart from an expected topology. Nevertheless, other explanations can account for these incongruent topologies, including reconstructions that do not accurately reflect the history of the gene due to problems such as the failure to account for rate-across-site variation, or covariance and biased amino acid composition across the tree [23]. Similarly, the history of the gene may be complex in ways that erroneously suggest lateral transfer even when the phylogeny is accurately reconstructed. For example, gene duplication events and differential extinction of the resulting paralogues across different lineages can lead to a tree that appears to reflect lateral transfer but that really describes the history of a duplicated gene.

Another emerging problem for the interpretation of lateral gene transfer is the observation of genes demonstrating punctate distributions. For eukaryotes, the term 'punctate distribution' has been used to refer to cases where two or more distantly related eukaryotic lineages possess closely related genes that are either not found in other eukaryotes, or are clearly different from other eukaryotic homologues [24]. This situation is in contrast to 'patchy distribution', a term that has been used to refer to genes with a limited distribution in both eukaryotes and prokaryotes [25]. Genes with a punctate distribution are significant because they have been interpreted in several different ways, each interpretation having its own important implications. On one hand, such distributions have been supposed to represent a single transfer to the common ancestor of two or more disparate lineages. The reconstruction of several large-scale relationships among eukaryotes (the so-called supergroups) has been partially based on the documentation of shared, rare characteristics such as gene fusions or indels in two or more lineages [26-28]. Shared lateral gene transfers have also been regarded in this way. Recent examples include the shared possession of a nanoarchaeal prolyl-tRNA synthetase in trichomonad and diplomonad flagellates [29] and a haloarchaeal tyrosyl-tRNA synthetase in opisthokonts [30]. In contrast, more complex distributions have been interpreted to represent multiple transfers or eukaryote-to-eukaryote transfers. These cases are also significant because detecting transfers between eukaryotes is made difficult by poor sampling of many lineages and poor resolution of many phylogenies, both of which impede the distinction between horizontal and vertical descent. For this reason, some eukaryote-to-eukaryote transfers have been argued based on the fact that the gene bears some special feature, such as an insertion, an accelerated rate of substitution, or even an origin from another lateral transfer event [18,24,29].

For any of these genes the possibility of ancient paralogy followed by selective loss or retention in diverse eukaryotes must also be considered. Even in such cases however, the origin of the gene may still ultimately be due to a lateral transfer event, whereas its distribution is due to other factors. Deciding between these interpretations involves balancing a variety of observations, including how widespread the gene in question is, how closely related the organisms that possess the gene are believed to be, whether other close relatives possess or lack the gene, and how convincingly a source lineage for the gene can be identified. In many cases we cannot answer these questions because data are lacking from a sufficient diversity of eukaryotes, such that it is impossible to conclude what might be the underlying cause. Moreover, it is impossible to say whether the factors resulting in punctate clades are common or rare: in many cases bacterium-to-eukaryote transfers have been inferred from data with seemingly simple distributions, but it is possible that some of these distributions only appear simple because of sampling deficiency.

Here we use EST data to evaluate what are apparently simple cases of lateral transfer of genes involved in core carbon metabolism. Surprisingly, we find in each instance a more complex, punctate distribution than suggested by the initial observations. In this study, we characterize protist homologues of three genes: ribulose-5-phosphate-3-epimerase (RPE), glyceraldehyde-3-phosphate dehydrogenase (GAPDH), and transketolase (TK). In the case of RPE, a transfer event has been described between a γ-proteobacterium related to *Pseudomonas *and a chlorarachniophyte [15]. Similarly, many lateral transfer events involving eukaryotic GAPDH have been described [15,21,31-34], including one isolated clade of diplonemid genes inferred to be have been transferred from a proteobacterium [33]. In the case of transketolase (TK), the chlorarachniophyte TK is not closely related to other eukaryotic homologues, but is very similar to homologues from Chlamydiales. For each of these genes, a single transfer from a bacterium to a eukaryote is evident from the relationship between the eukaryotic and a particular subgroup of bacteria, but we show here that the distribution within eukaryotes suggests a more complicated history. In all three cases we find homologues in other eukaryotes that are only distantly related to the organism in which the gene was first found. How the complex distribution of these genes arose is uncertain, but these examples indicate that such a punctate pattern of presence is more common than previously thought, leading us to suggest that other apparently simple cases of lateral transfer may be more complex than they appear.

## Results & Discussion

### Ribulose-5-Phosphate-3-Epimerase

**Figure 1:**
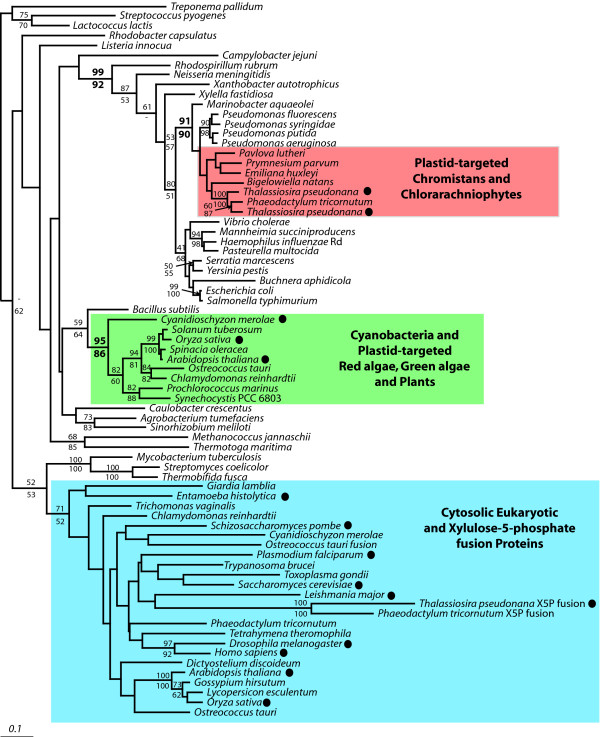
**Bayesian phylogenetic tree of ribulose-5-phosphate-3 epimerase (RPE)**. The tree was inferred from 183 amino acid characters with branch lengths estimated using PROML. Bootstrap values > 50% are shown. Values shown above a node correspond to PHYML bootstrap support, those below a node correspond to WEIGHBOR support. Eukaryotic sequences are enclosed in boxes where blue corresponds to the major clade of cytosolic proteins, green corresponds to plastid-targeted proteins, and red corresponds to bacterium-derived genes. Filled circles adjacent to taxon names indicate that a complete genome is available from this organism.

Ribulose-5-phosphate epimerase (RPE) catalyzes the bidirectional conversion of ribulose-5-phosphate to xylulose-5-phosphate both in the Calvin cycle in the plastid of photosynthetic eukaryotes and in the cytosolic pentose-phosphate pathway of both photosynthetic and non-photosynthetic eukaryotes. Phototrophic eukaryotes therefore have two forms of this enzyme: the plastid-targeted form in red algae, green algae and plants is cyanobacterial, whereas the cytosolic form is related to that of non-photosynthetic eukaryotes, as expected (Figure 1). The relationships among cytosolic epimerases of eukaryotes are poorly resolved, with only recently diverging groups such as vascular plants and metazoa recovered with strong bootstrap support.

Previously, it has been shown that the plastid-targeted RPE of the chlorarachniophyte *B. natans *is not related to other plastid-targeted or even cyanobacterial genes, as one would expect, but is instead closely related to the γ-proteobacterial genus *Pseudomonas *[15]. By increasing the sampling of RPE from EST and genomic data of other protists, a similar proteobacterial RPE sequence was found in six different chromalveolate genomes. Phylogenetic analysis confirmed that RPE genes from the haptophytes *Emiliania huxleyi*, *Prymnesium parvum *and *Pavlova lutheri *are all of the γ-proteobacterial type, as are those from the diatoms *Thalassiosira pseudonana *and *Phaeodactylum tricornutum *(Figure 1). A fragment of a highly similar gene was also identified in the haptophyte *Isochrysis galbana *(GenBank accession EC139053). It was too short to be included in the analysis, but preliminary trees confirmed it was closely related to the other haptophyte RPE genes (not shown). Relationships between the chromalveolate and *B. natans *genes (here designated RPE-γ) are unresolved, but collectively they form a strongly-supported group with γ-proteobacterial homologues, and more specifically the relationship with pseudomonads and alteromonads remains well supported. The RPE-γ from *B. natans *was reported to have a truncated N-terminal leader, suggesting it is plastid-targeted [15]. Evidence for this clade consisting of plastid-targeted proteins also comes from *P. tricornutum *and *E. huxleyi *RPE-γ sequences, that have full-length leaders predicted to encode signal peptides at the N-terminus, which is a characteristic of plastid-targeted proteins in these organisms. The *P. parvum*, *P. lutheri *and *T. pseudonana *RPE-γ sequences also all have truncated N-terminal leaders, further suggesting that this entire clade of proteobacterium-derived RPE proteins is plastid-targeted. Unlike the RPE of *B. natans *which has been demonstrated to be a bacterial gene in a eukaryotic genome by the presence spliceosomal introns, and the diatoms RPEs which have been assembled in to a eukaryotic genome, the possibility of bacterial contamination in the *P. parvum*, *P. lutheri *and *E. huxleyi *ESTs cannot be formally ruled out, but is very unlikely for several reasons relating to how the sequences were generated (see Methods for details).

The two diatom genomes encode another RPE that branches with the cytosolic homologues of other eukaryotes, but there is no evidence in any of the five chromalveolates for a cyanobacterium-derived, red algal-type RPE gene that would be expected to operate in the plastids of these organisms. This search included the complete genome sequence of *T. pseudonana *and the nearly complete sequence of the *P. tricornutum *genome, as well as the extensive EST databases that have been generated for the three haptophytes. It would appear that the ancestral, cyanobacterium-derived RPE is absent from all of these chromalveolates. A second, bacterial-type RPE was found in *I. galbana *(GenBank accession EC141129), but it was not found to be related to plastid or other eukaryotic homologues.

The origin of the γ-proteobacteria-like RPE gene in eukaryotes and its distribution must be considered separately. The origin of the RPE-γ gene is addressed by the strong support for the eukaryotic genes being sister to a specific and taxonomically narrow group of bacteria, the pseudomonads. This result argues for a relatively recent origin by lateral gene transfer from the pseudomonads to eukaryotes. The distribution of this gene, however, is more complicated because the eukaryotes that contain RPE-γ are not all closely related. Evidence exists that haptophytes and diatoms are both members of the supergroup Chromalveolata, but chlorarachniophytes belong to a completely different supergroup, Rhizaria [35]. Explaining this complex distribution by paralogy would be relatively simple if the enzyme were cytosolic: one would then propose that rhizaria and chromalveolates were specifically related and that many of the constituent lineages of these two supergroups had lost the enzyme (considering only complete or nearly complete genomes, this would include apicomplexa, *Perkinsus *and ciliates). However, the fact that RPE-γ is targeted to the plastid substantially complicates this interpretation because the *B. natans *plastid is derived from a green alga whereas the chromalveolate plastids are derived from a red alga. The plastid-targeted RPEs of both green algae and red algae are cyanobacterial (Figure 1) and in neither group has the proteobacterial RPE-γ type been found in available complete genome sequences. Accordingly, if the plastid RPEs in chromalveolates and chlorarachniophytes were derived from a common ancestor, the proteobacterial type would have had to coexist with the cyanobacterial type in an ancestor of red and green algae, with subsequent diversification involving a complex pattern of reciprocal losses not only in rhizarians and chromalveolates, but also in red and green algae. Alternatively, if the enzyme was a cytosolic RPE in a hypothetical common ancestor of chlorarachniophytes, haptophytes, and diatoms, then it would have had to have taken over plastid function twice independently, in addition to reciprocal losses. Both of these explanations are very complicated and invoke higher-order relationships among eukaryotes that are not known. In addition, the close specific relationship between the pseudomonad and the eukaryotic RPE-γ sequences is more suggestive of a recent origin by lateral transfer than an ancient origin. Taken together, the simplest explanation for the current distribution is that RPE-γ was transferred from a pseudomonad to an undefined eukaryotic lineage and then transferred between two eukaryotic lineages (the direction cannot be inferred from the phylogeny because the topology of rhizarian and chromalveolate RPEs is not resolved).

In the course of this study, we also observed an interesting gene fusion event involving the cytosolic RPE of the diatoms *T. pseudonana *and *P. tricornutum *and the prasinophyte green alga *Ostreococcus tauri*. In these three organisms, the cytosolic-type RPE is found as a xylulokinase-RPE fusion-protein. In *P. tricornutum *and *O. tauri *the two proteins are part of an uninterrupted ORF, whereas in the *T. pseudonana *genome, the xylulokinase and RPE are in different reading frames, although this apparent frameshift is most likely due to the presence of an unannotated intron. Xylulokinase catalyses the phosphorylation of xylulose to xylulose-5-phosphate for entry into the pentose phosphate pathway. This reaction occurs immediately prior to the ribulose-5-phosphate epimerase reaction, raising the intriguing possibility that the fusion protein may catalyze both reactions. Interestingly, the N-termini of the *P. tricornutum *and *T. pseudonana *RPE proteins also encode a predicted signal peptide, suggesting that this fusion protein may be targeted to the plastid. *P. tricornutum *and *O. tauri *also encode canonical cytosolic RPEs related to other cytosolic isoforms. Gene fusion events involving carbon metabolic enzymes have been reported from other algae, notably those involving GAPDH and enolase in dinoflagellates [36], and between triose phosphate isomerase and GAPDH in the mitochondria of heterokonts [37]. Whether the fusions had a common origin or arose independently is not clear: *O. tauri *is a green alga whereas diatoms have plastids derived from red algae. The fusion genes are not demonstrably related (Figure 1), suggesting perhaps that the fusion arose twice independently.

### Glyceraldehyde-3-Phosphate Dehydrogenase

**Figure 2:**
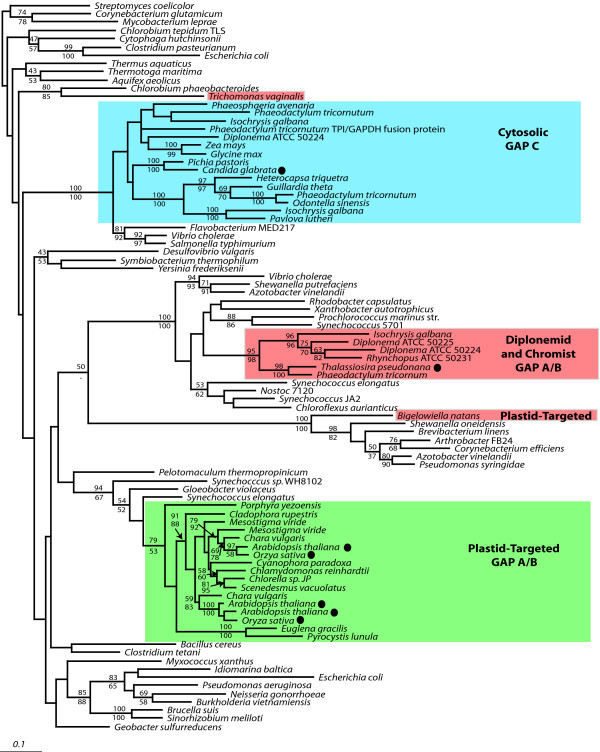
**Bayesian phylogenetic tree of glyceraldehyde-3-phosphate dehydrogenase (GAPDH)**. The tree was inferred from 278 amino acid characters with branch lengths estimated using PROML. Bootstrap values > 50% are shown. Values shown above a node correspond to PHYML bootstrap support, those below a node correspond to WEIGHBOR support. Eukaryotic sequences are enclosed in boxes where blue corresponds to the major clade of cytosolic proteins, green corresponds to plastid-targeted proteins, and red corresponds to bacterium-derived genes (the *B. natans *plastid targeted GAPDH is also bacterium-derived and is coloured red). Filled circles adjacent to taxon names indicate that a complete genome is available for this organism.

Glyceraldehyde-3-phosphate dehydrogenase (GAPDH) catalyzes the bi-directional conversion of glyceraldehyde-3-phosphate to 3-phosphoglycerate in both glycolysis and the Calvin cycle. GAPDH has been extensively sampled from eukaryotes and bacteria, revealing many cases of lateral transfer and paralogy. Bacteria and plastids canonically use a class of enzyme known as GapA/B, whereas the typical eukaryotic cytosolic GAPDH is called GapC. The ancient evolution of this family is complex as several gene duplications have taken place and GapC as a whole has been suggested to be derived by lateral transfer [38,39]. These ancient events remain uncertain, but several eukaryotic groups also have genes that are clearly from the GapA/B class: these are not derived from the plastid endosymbiont but are considered to have originated by relatively recent lateral transfer [15,21,31,40]. One of these cases is the divergent class of GapA/B (here designated GapA/B*) previously found only in diplonemids, heterotrophic relatives of kinetoplastids [33]. Once again, however, with increased sampling we find the same class of GAPDH in haptophytes (*I. galbana*) and diatoms (*T. pseudonana *and *P. tricornutum*) (Figure 2). EST data from the haptophyte *E. huxleyi *also include three transcripts encoding a similar GapA/B* gene (GenBank accessions CX777351, CX776621, and EG034112), but these sequences are too short to be included in the phylogeny. As noted above, heterokonts and haptophytes are thought to be members of the same supergroup, chromalveolates, but they are not closely related to diplonemids, which are members of the excavates. Nevertheless, the GapA/B* sequences from all three groups form a strongly supported clade, which in turn branches within a eubacterial group consisting of proteobacteria and cyanobacteria, as found previously for diplonemids alone [33]. The chromalveolate GapA/B* sequences are paraphyletic in this analysis, as the diplonemids share a robust sister relationship with *I. galbana *to the exclusion of diatoms. The *I. galbana *GAPDH is not full length, but the N-termini of the two diatom sequences are of comparable length to GapA/B in bacteria and do not encode a predicted signal peptide, so these proteins are likely cytosolic.

The distribution of GapA/B* bears many similarities to that of RPE-γ : the gene is found in distantly related clades (chromalveolates and diplonemids), each of which has relatives that lack it. Also in common with RPE-γ, the close relationship between the eukaryotic GapA/B* genes and their bacterial sisters, together with their distant relationship to canonical eukaryotic GapC genes, indicates that the eukaryotic homologues originated by lateral transfer from a bacterium. However, this conclusion does not address present-day distribution of GapA/B* in eukaryotes. If there was a single transfer to the ancestor of chromalveolates and diplonemids, then the gene must have been lost in many of the relatives of these two groups (considering only taxa where complete or nearly complete genomes are known, this includes apicomplexa, *Perkinsus*, ciliates, trypanosomatids, and perhaps *Giardia *and *Trichomonas*). However, given the large number of lateral transfer events already known to have involved GAPDH, including one between two eukaryotes [21], the current narrow range of taxa possessing the GapA/B* gene suggests instead that it was transferred from a bacterium relatively recently and subsequently spread to other eukaryotes by eukaryote-to-eukaryote transfers. The branching order between the eukaryotic GapA/B* genes is well supported, and at face value this result suggests that there were either multiple transfers between eukaryotes or that the gene originated in chromalveolates and was transferred to diplonemids.

### Transketolase

Transketolase (TK; glycoaldehydetransferase) catalyzes the reversible transfer of a C_2 _unit between two 5-carbon sugars, producing a 3-carbon sugar and a 7-carbon sugar, or between a 4-carbon sugar and a 5-carbon sugar, producing a 6-carbon sugar and a 3-carbon sugar. TK functions in the cytosol of non-photosynthetic eukaryotes, where it is involved in reactions of the classic pentose-phosphate pathway. It is also found in the plastids of algae and plants, where it functions in the Calvin cycle as well as the reversible branch of the pentose-phosphate pathway. In at least some plants, an additional cytosolic isoform of TK, related to the plastid form, exists [41].

**Figure 3:**
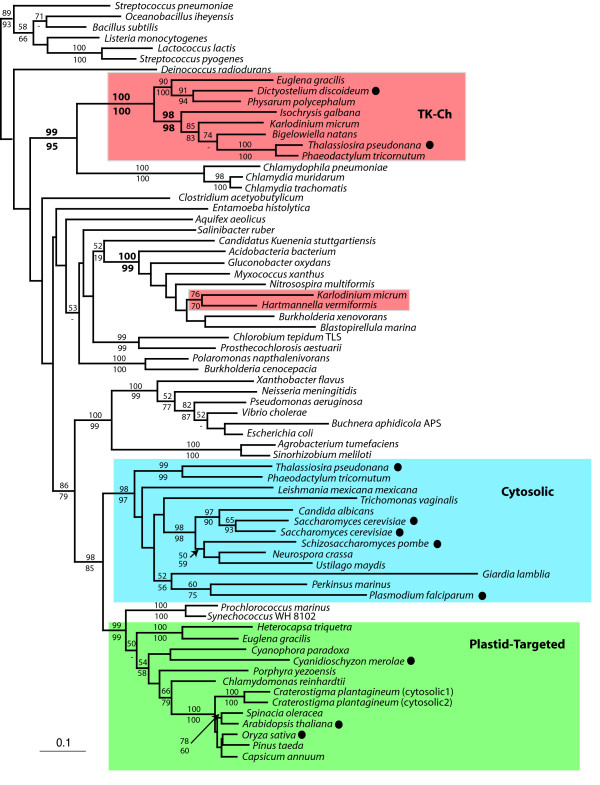
**Bayesian phylogenetic tree of transketolase (TK)**. The tree was inferred from 473 amino acid characters with branch lengths estimated using PROML. Bootstrap values > 50% are shown. Values shown above a node correspond to PHYML bootstrap support, those below a node correspond to WEIGHBOR support. Eukaryotic sequences are enclosed in boxes where blue corresponds to the major clade of cytosolic proteins, green corresponds to plastid-targeted proteins, and red corresponds to bacterium-derived genes. Filled circles adjacent to taxon names indicate that a complete genome is available from this organism. Multiple isoforms of TK are present in the diatom genomes, both a *Chlamydia *type TK as well as a eukaryotic form of this enzyme are present. Two types of TK are present in *Euglena *as well, a form related to the plastid TK of other eukaryotes as well as a *Chlamydia *type.

TK exhibits a complex phylogenetic distribution across different groups of eukaryotes. Metazoa and ciliates have a highly divergent form of TK characterized by many gaps and deletions; we have not included these sequences in our data set because they are difficult to align with other TKs. Most eukaryotic cytosolic TKs belong to a more conserved group that is widespread and constitutes a single well-supported clade (Figure 3). Similarly, most plastid-targeted TKs have robust cyanobacterial affinities, as expected, although one with an unusually close relationship to the cytosolic clade described above. The phylogeny within the plastid-targeted clade is generally not well resolved; however, one interesting exception are the plastid-targeted TKs from the euglenid *Euglena gracilis *and the dinoflagellate *Heterocapsa triquetra*, which form a very strongly supported branch in all analyses and which fall at the base of the plastid-targeted clade with strong support in analyses of the full protein (Figure 3, 'Plastid-targeted'). Euglenids and dinoflagellates are not closely related and their plastids are derived from green and red algae, respectively. That these two sequences branch together is therefore unusual and reminiscent of a proposed transfer of a plastid-targeted GAPDH between euglenids and dinoflagellates [36].

**Figure 4:**
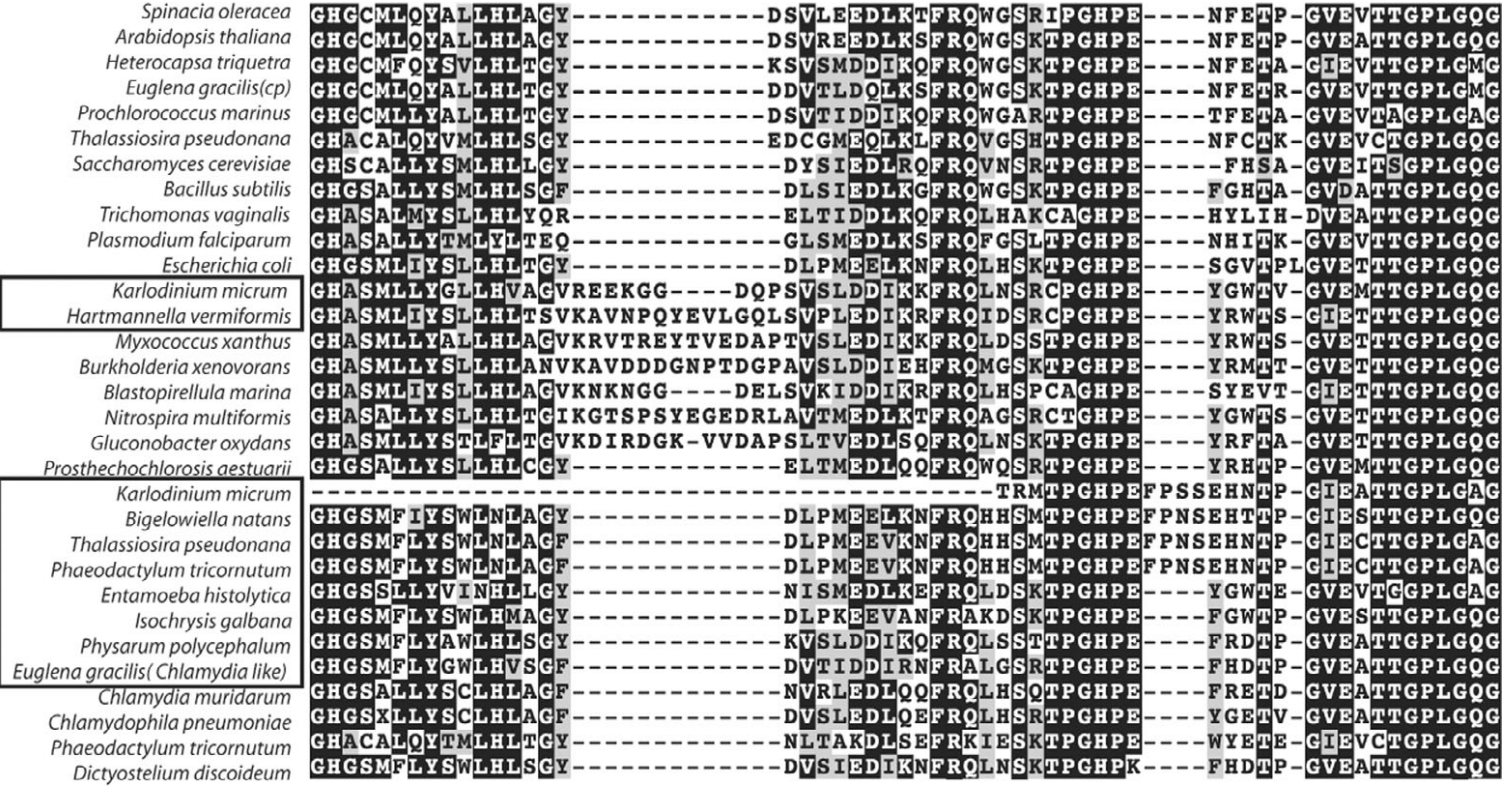
**Transketolase alignment flanking a 4-amino acid insertion present in diatom, dinoflagellate and chlorarachniophyte TK-Ch genes**. A nearby 11- to 15-amino acid insertion characterizes a mixed group of CFB bacteria and proteobacteria as well as the amoebozoan *Hartmannella vermiformis *and the dinoflagellate *Karlodinium micrum*. Eukaryotic TKs putatively derived from lateral transfer events are surrounded by black squares (TK-Ch being the lower box).

The most interesting clade, however, comprises several eukaryotic TKs that are unrelated to either the major cytosolic clade or plastid-targeted clade, but instead are closely related to the bacterial group Chlamydiales (Figure 3). This clade (which we term TK-Ch) includes not two, but several distantly related groups of eukaryotes. The chromalveolates are most heavily represented, including the diatoms *T. pseudonana *and *P. tricornutum*, the haptophyte *I. galbana*, and the dinoflagellate with a haptophyte plastid, *Karlodinium micrum*. Also included are the amoebozoans *Dictyostelium discoideum *and *Physarum polycephalum*, the excavate *E. gracilis*, and the rhizarian *B. natans*, an assemblage that accounts for four of the five eukaryotic supergroups (the exception being plants). In addition, more highly truncated ESTs were found from several other chromalveolates, specifically the haptophyte *P. parvum *and the cryptomonad *Guillardia theta*, as well as ESTs representing another copy of the gene from *K. micrum*. These sequences were too short to include in the analysis shown in Figure 3, but in phylogenies restricted to the 3' end of the gene they consistently branch within this clade with high support (not shown). Amoebozoans and *E. gracilis *consistently form a strongly supported group, as do the chromalveolates and *B. natans*. Within the latter clade, *I. galbana *occupies a basal position in analyses based on the nearly complete amino acid sequence, and this result is strongly supported both by bootstrap values and by the presence of a unique conserved 4-amino acid insertion in the *K. micrum*, *B. natans *and diatom TK-Ch sequences that is absent from *I. galbana *TK-Ch (Figure 4).

We cannot determine whether the TK-Ch proteins of *B. natans *and *K. micrum *are cytosolic or plastid-targeted as they are N-terminally truncated. However, the *E. gracilis *and two diatom sequences are comparable in length at their N-terminus to bacterial TKs and cytosolic TKs of other eukaryotes, and neither is predicted to encode a signal peptide. The transketolase of *Isochrysis galbana *encodes a long n-terminal leader predicted to encode a signal peptide, suggesting that this transketolase may be plastid-targeted in *I. galbana*. Altogether, the evidence suggests that these TK-Ch proteins are plastid-targeted in some organisms such as the haptophyte *I. galbana*, but cytosolic in other photosynthetic organisms such as the diatoms *P. tricornutum *and *T. pseudonana *and the euglenid *E. gracilis*.

In addition to this group, there are also a few eukaryotic sequences that fall outside any of the TK groups characterized so far. In particular, the amoebozoan *Hartmannella vermiformis *and the dinoflagellate *K. micrum *both have EST-predicted TKs related to a proteobacteria-planctomycetes-CFB bacterial clade (a relationship that is further supported by an insertion: Figure 4), and that constitute a weakly supported clade (Figure 3). The *Entamoeba histolytica *TK, on the other hand, branches outside any eukaryotic clade but does not show an affinity to any other group.

Distinguishing between lateral transfer and paralogy in the TK case is a more complex problem than in the RPE and GAPDH situations considered above, because the diversity of eukaryotes with TK-Ch is much greater. At the same time, this breadth makes the TK case potentially much more interesting, and significant. This wide distribution makes a stronger case for paralogy – that this gene represents an ancient eukaryotic paralogue present in the last common ancestor of these groups. This argument implies that the gene was lost in close relatives of extant organisms that contain TK-Ch, which is a substantial qualification because a large number of losses would be required: considering only groups where genome sequences are complete or nearly so, this would include animals, fungi, *Entamoeba*, apicomplexa, ciliates, *Perkinsus*, kineteoplastids, *Giardia *and *Trichomonas*. The specific relationship between the eukaryotic TK-Ch and Chlamydiales TK sequences is also difficult to reconcile with such an ancient origin, suggesting instead that the corresponding gene may have originated more recently by lateral gene transfer from an ancestor of the Chlamydiales group.

If, in fact, the TK-Ch gene was transferred to an ancient ancestor of most or all eukaryotes, no eukaryote-to-eukaryote transfers need be invoked. However, this strict interpretation runs into difficulties when the phylogeny within the TK-Ch clade is considered. If no between-eukaryote transfer had occurred, then the supergroups should be monophyletic or at least unresolved. This is not the case, since the rhizarian *B. natans *branches within the chromalveolates with strong support (Figure 3), and its relationship to *K. micrum *and the diatoms is further supported by the shared insertion (Figure 4). To explain these observations without lateral transfer, it would be necessary to propose additional cases of paralogy arising since the gene originated in eukaryotes. The alternative explanation is that the *B. natans *gene is derived from lateral transfer from another eukaryote, which is consistent with the observation that nearly a dozen other *B. natans *genes have been derived from other phototrophs by lateral transfer [15]. By extension, there is no reason to exclude the possibility that there have been other transfers between eukaryotes (the present distribution could be achieved with as few as three transfers), which would also explain the punctate distribution without having to argue for loss in close relatives. This interpretation provides the simplest explanation of the current data. If eukaryote-to-eukaryote gene transfer is the underlying mechanism by which TK-Ch came to exhibit its present distribution, then the resulting pattern is second in complexity only to that of the previously described case of a novel elongation factor-like GTPase, EFL [24].

## Conclusion

We describe three examples where the phylogeny of a carbon metabolic enzyme at first appeared to indicate a simple case of bacterium-to-eukaryote lateral gene transfer, but where greater sampling has shown the situation to be considerably more complex. In all three cases other eukaryotes with the same bacterial gene have been discovered, and in each case these eukaryotes are only distantly related to one another at the organismal level. The first important point to note here is that these observations emerged only with an increased sampling of eukaryotic molecular diversity, implying that the distributions reported here are likely to change further as taxon sampling becomes even more comprehensive. However, the distribution can only change in one direction – toward greater complexity. It is possible that these genes will ultimately be found in such a large sample of eukaryotes we will ultimately conclude that they represent ancient paralogues whose distribution is mostly due to gene loss; on the other hand, considering the high frequency of the absence of these particular sequences in current data, it seems more likely they will continue to be rare and to exhibit a punctate distribution. In the case of the EFL GTPase further sampling has revealed additional organisms that possess the corresponding gene [42,43]; nevertheless, EFL remains far less common than its counterpart, EF-1α.

A second noteworthy point is that we often contrast lateral gene transfer and lineage sorting as two contradictory possibilities, whereas they work concurrently. If a new gene arrives in a lineage by lateral transfer, some descendents may keep it and others may lose it, resulting in an apparently complex distribution. Distinguishing this pattern from that resulting from serial transfers is difficult and may well be impossible in certain circumstances; however, we can still weigh the observations in favour of one possibility over the other. In particular, lineage sorting is probably more likely in cases where a complex distribution of presence and absence is found in closely related species, whereas serial transfers are more likely when the time frame is significantly longer. In the three cases described here, the time frame is very long indeed, and there is evidence from the internal phylogenies for eukaryote-eukaryote transfer. The significance of this finding extends beyond these three genes, to the process of transfer between eukaryotes in general. Currently, few cases of such transfers are known because they are difficult to detect in the absence of better sampling than is currently available for most genes. The results reported here use an unusual feature of the gene, its origin from bacteria, as a flag to draw attention to subsequent transfers; however, there is nothing to indicate that the same process could not be occurring in many other genes where it is not as evident because of the lack of such flags.

## Methods

### Characterization of new sequences

Using TBestDB [44], clones corresponding to TK were identified in EST projects from *Euglena gracilis *(12 ESTs for the chloroplast-targeted isoform and 3 ESTs for the TH-Ch isoform)*, Bigelowiella natans *(3 ESTs),*Hartmannella vermiformis *(9 ESTs), *Karlodinium micrum *(4 ESTs for the TK-Ch isoform and 1 EST for the CFB group isoform), *Isochrysis galbana *(7 ESTs) and *Physarum polycephalum *(18 ESTs). Clones corresponding to RPE were identified from *Pavlova lutherii *(3 ESTs). ESTs were re-sequenced to obtain full-length assemblies wherever possible. Putative full-length transcripts were assembled in this way for TK from *E. gracilis*, *P. polycephalum *and *H. vermiformis*. 5'-Truncated assemblies were obtained from *K. micrum *and *I. galbana*. The 5' end of *B. natans *TK was obtained through PCR amplification using degenerate TK primers (CGCGACTACAGGCCCNYTNGGNCARGG and GCGCAAGGCGAACWSNGGNCAYCCNGG) and specific primers based on the EST sequences (CTCTCCAACACCGATAGAATCATGAGTC and GCCTTGTACCGGGTGATGACATCCTCAG). A PCR product from *I. galbana *with similarity to bacterial GAPDHs was obtained through PCR using degenerate primers (CCAAGGTCGGNATHAAYGGNTTY and CGAGTAGCCCCAYT CRTTRTCRTACCA). All PCR products were cloned using the TOPO TA vector (Invitrogen) and multiple copies were sequenced on both strands. New sequences were deposited in GenBank as accessions EF216678-EF216685, EF221881 and EF375722. RPE genes were also identified in EST projects from *Emiliania huxleyi *(7 ESTs) and *Prymnesium parvum *(1 EST). Homologues of all three genes were also identified in the completed genomes of *Thalassiosira pseudonana *[45] and *Phaeodactylum tricornutum *[46].

### Sequence analyses

For organisms possessing a complex plastid, the probable plastid localization of all full length sequences was evaluated using SIGNALP v. 3.0 [47] and leader sequences were manually scanned for characteristics expected of transit peptides for the group in question.

New sequences were aligned to homologues from public databases using CLUSTAL X, and manually edited using MacClade 4.07. Publicly available sequences used in alignments were downloaded from nr GenBank, ESTdb, or from complete eukaryotic genome databases. All sequences found to be closely related to the eukaryotic genes in question were included, along with a representative selection of other genes. Genes that were closely related to some other gene but not the eukaryotic genes in question were generally excluded, as were some genes that were highly divergent (e.g. the animal TK). Positions that were not clearly homologous were excluded, resulting in a full TK alignment with 473 characters, and a reduced alignment with 212 characters that was also used to include those sequences with missing 5' sequence. The RPE alignment consisted of 183 alignable characters and the GAPDH alignment comprised 278 alignable characters. Alignments of all three genes are available upon request from PJK.

While the possibility that several of these sequences are artifacts from a bacterial genome exists, several lines of evidence argue against this. ESTs encoding the proteobacterial-like RPE from haptophytes branch together (weakly) to the exclusion of all bacterial sequences. This suggests that if these sequences arose through bacterial contamination, the source of the contamination must have been similar in each library. Also, multiple ESTs encoding RPE in *P. lutheri *and *E. huxleyi *are present in these two libraries (two and seven respectively), indicating that if this RPE is due to a bacterial contaminant, the contamination must be highly represented in these libraries. Lastly, the RPE sequence of *E. huxleyi *encodes an N-terminal extension with a predicted signal peptide, a hallmark of plastid-targeted proteins in eukaryotes with complex plastids. Similarly, a contamination artefact cannot be precluded for the *K. micrum *and *H. vermiformis *TKs that branch within a clade consisting of CFB bacteria, proteobacteria and planctomycetes. Like the RPEs of the haptophytes, we would have to conclude that the contamination arose from a similar source since the two sequences branch together with moderate support in our analysis. The *K. micrum *TK is encoded by a single EST, though the *H. vermiformis *TK is encoded by nine ESTs suggesting that if this *H. vermiformis *sequence is a contaminant, it is highly represented among the *H. vermiformis *ESTs.

Phylogenetic analyses were carried out using maximum likelihood, distance and Bayesian methods. Maximum likelihood phylogenies were performed using PHYML 2.4.4 [48] with the WAG substitution matrix and site rate distribution modeled on a discrete gamma distribution with 4 rate categories and one category of invariable sites. The estimated alpha parameters were 1.154, 1.156, and 1.419 and the estimated proportions of invariable sites were 0.067, 0.050, and 0.101 for RPE, GAPDH, and TK, respectively. Bayesian analysis of trees was performed using MrBayes 3.0b4 [49] run using the WAG substitution matrix and a gamma distribution with 4 rate categories and one category of invariable for 1,000,000 generations with sampling every 1,000 generations. Distance analyses were performed using TREE-PUZZLE 5.2 [50] with four rate categories and 1 category of invariable sites. Alpha parameters inferred by TREE-PUZZLE were 0.89, 1.04 and 1.21 and the estimated proportions of invariable sites were 0.04, 0.05 and 0.10 for RPE, GAPDH and TK respectively. WEIGHBOR 1.0.1a was used to reconstruct distance trees Bootstrapped distance matrices were generated using PUZZLEBOOT [51] with alpha parameter and proportion of invariable sites estimated using TREE-PUZZLE 5.2.

## Authors' contributions

MBR identified and characterized new genes, performed phylogenetic analyses and drafted the manuscript. RFW and JTH characterized new genes. DGD and MWG contributed to interpretation and helped to draft the manuscript. PJK conceived of the study, and participated in its design and coordination and helped to draft the manuscript. All authors read and approved the final manuscript.
